# Hypovirulence-Based Biocontrol: Mechanisms, Applications, and Prospects for Sustainable Crop Protection

**DOI:** 10.3390/plants15213272

**Published:** 2026-10-23

**Authors:** Chang-Jie Jiang, Yao Sun, Ruiming Zhang, Yanan He, Xinqiong Liu, Xianzhi Xie

**Affiliations:** 1 Shandong Key Laboratory of Genome Modification Technology and Germplasm Innovation, Institute of Wetland Agriculture and Ecology, Shandong Academy of Agricultural Sciences, Jinan 250100, China; cypa44@hotmail.com (C.-J.J.); sunyao0525@gmail.com (Y.S.); sdauzbzrm@163.com (R.Z.); hyn2013nice@163.com (Y.H.); 2 College of Life Science, South-Central Minzu University, Wuhan 430074, China

**Keywords:** hypovirulence, attenuated-pathogen biocontrol, mycovirus, biological control, plant immunity, defense priming, cross-protection

## Abstract

Hypovirulence, defined as a reduction in a pathogen’s capacity to cause disease, can arise naturally or be induced by mycovirus infection, genetic mutation, gene silencing, or other molecular interventions. As an environmentally compatible alternative or complement to chemical pesticides, hypovirulence offers a promising strategy for sustainable crop disease management. A central principle underlying effective hypovirulence-based systems is the balance between attenuation and biological competence: pathogenicity must be sufficiently reduced, while the hypovirulent agent retains the capacity to survive, colonize, persist, or confer protection. Disease suppression can operate through four major, often interacting mechanisms: population-level spread of transmissible hypovirulence determinants, direct impairment of pathogen physiology and virulence traits, activation or priming of host defenses and beneficial endophytic interactions, and competitive exclusion or viral cross-protection. These mechanisms may offer high specificity and reduced non-target impacts, although ecological safety requires system-specific experimental validation; they also provide modes of action distinct from those of conventional pesticides and are suitable for integration into integrated pest management (IPM) programs. This review synthesizes current evidence from fungal, bacterial, and viral pathosystems, explicitly distinguishes laboratory and greenhouse proof-of-concept studies from repeated field evidence, and discusses major biological and operational constraints. We further examine emerging technologies while distinguishing experimentally demonstrated applications from prospective research directions that may improve the reliability, applicability, and scalability of hypovirulence-based biocontrol.

## 1. Introduction

Global crop production is under increasing pressure from plant pathogens and other pests, which cause substantial losses in major food crops worldwide [1]. Climate change further exacerbates this challenge by expanding pathogen geographic ranges and accelerating disease cycles [2]. Conventional management has relied heavily on chemical fungicides, bactericides, and resistant cultivars; however, the emergence of resistant pathogen populations, regulatory restrictions on agrochemicals, and increasing public concern over environmental and food-safety impacts have driven the search for sustainable alternatives [3]. Consequently, biological control, broadly defined as disease suppression through living organisms or their biological activities, has become an important component of integrated pest management (IPM) strategies [4].

Among biological approaches, attenuated pathogens occupy a distinctive position because they exploit biological functions of the pathogen itself. A weakened strain may retain enough competence to persist, colonize the host or infection niche, compete with virulent strains, transmit an attenuating element, or stimulate host defense, while causing little disease. This idea has deep historical roots: prior exposure to weakened pathogens was reported to reduce subsequent disease as early as 1901 [5,6], and the discovery of virus-associated recovery from chestnut blight established fungal hypovirulence as a major model for biological control [7].

The practical value of attenuated-pathogen systems lies in this balance between reduced virulence and retained biological competence. Compared with broad-spectrum pesticides, such systems can be highly specific, compatible with the target pathogen’s ecological niche, and mechanistically distinct from conventional fungicide targets. In some cases, transmissible mycoviruses can spread the attenuated phenotype through pathogen populations, whereas in others, non-viral attenuated strains or mild viruses suppress disease by colonization, immune priming, or cross-protection [8,9,10,11].

This review uses classical fungal hypovirulence as the conceptual foundation but extends the comparison to functionally related attenuated bacterial and viral systems. The categories are explicitly functional and are not intended to imply mechanistic equivalence among fungal mycovirus-mediated hypovirulence, non-viral fungal attenuation, bacterial competition or defense induction, and mild-virus cross-protection. Previous reviews have primarily synthesized mycovirus diversity, molecular biology, or the transition from mycovirus discovery to fungal disease control [8,9,12,13,14,15]. Here, we apply a structured cross-pathosystem framework based on three comparative axes: (i) the protective competence retained after attenuation, (ii) the strength and setting of the supporting evidence (laboratory, greenhouse, field, or sustained deployment), and (iii) the principal barrier to translation. The aim is therefore not simply to catalogue weakened pathogens, but to explain why some attenuated agents progress toward practical biocontrol whereas others remain experimental.

## 2. Definition and Origins of Hypovirulence

### 2.1. What Is Hypovirulence?

Hypovirulence is formally defined as a significant reduction in the disease-causing capacity of a pathogenic microorganism relative to the typical virulence of its species [8,16]. Unlike avirulence, which implies complete or near-complete loss of pathogenicity in a specific host context, hypovirulence is quantitative and can range from mild attenuation to near-complete loss of aggressiveness. Because the term has been used most extensively in fungal pathology, this review treats fungal hypovirulence as the core concept and considers avirulent bacterial strains and mild plant viruses as functionally related attenuated-pathogen systems rather than mechanistically equivalent forms of hypovirulence. Their inclusion is justified by a shared functional logic: reduced disease-causing capacity combined with retained competence for colonization, competition, transmission, immune stimulation, or cross-protection.

### 2.2. Major Mechanisms of Hypovirulence

**Figure 1:**
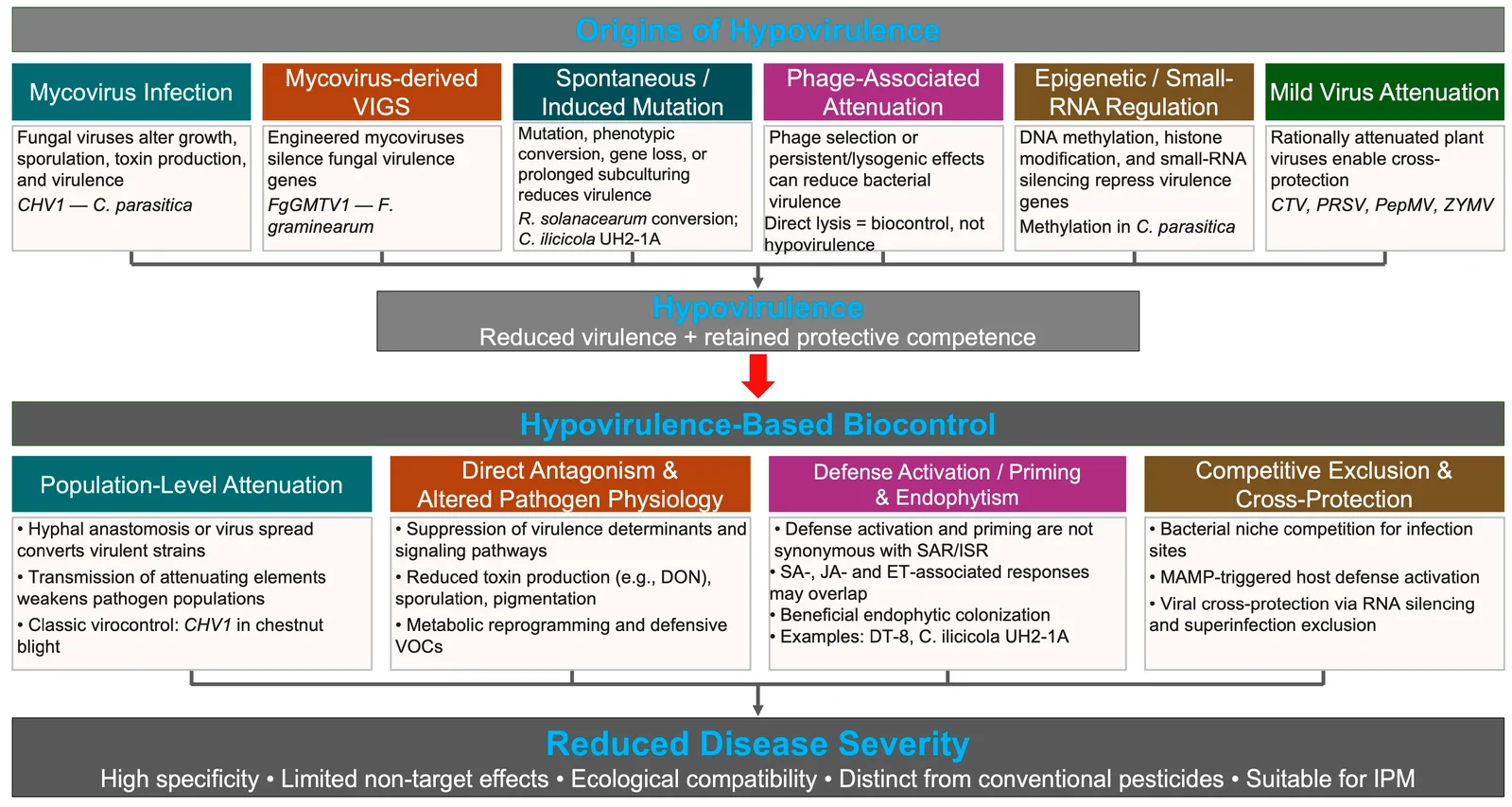
Schematic illustration of hypovirulence and hypovirulence-based biocontrol. Diverse routes of attenuation—including mycovirus infection, mycovirus-derived virus-induced gene silencing (VIGS), spontaneous or induced mutation, phage-associated bacterial attenuation, epigenetic or small-RNA regulation, and mild-virus attenuation—can generate weakened pathogen states. Direct lytic phage killing is biological control by pathogen removal and is not included as hypovirulence sensu stricto. The key conceptual requirement is an attenuation–competence balance: disease-causing capacity must be reduced while sufficient biological competence is retained for transmission, colonization, competition, immune stimulation, or superinfection exclusion. These protective capacities reduce disease through four complementary functional levels: population-level attenuation, direct physiological suppression of pathogens, plant defense activation/priming and beneficial endophytism, and competitive exclusion or viral cross-protection. Potential advantages such as high specificity and reduced non-target impacts are system-dependent and require experimental validation of ecological safety.

Hypovirulence can arise naturally or be generated through mycovirus infection, mutation, gene silencing, phage-associated attenuation, epigenetic regulation, or deliberate viral attenuation. These routes are mechanistically distinct. In particular, direct lytic phage killing is biological control by pathogen removal and is not hypovirulence sensu stricto; only phage-associated states in which bacterial viability is retained while pathogenicity is reduced are considered here as attenuation. The contents discussed in this section are summarized in Figure 1.

#### 2.2.1. Mycovirus Infection

Mycovirus infection is the best-studied origin of hypovirulence. Mycoviruses can alter fungal growth, sporulation, toxin production, stress tolerance, and virulence [9,12,13,16]. Many lack a conventional extracellular phase and are transmitted mainly through hyphal anastomosis or spores, although exceptions exist [8,17,18]. When virus infection reduces fungal virulence without eliminating host survival or transmission, the infected strain can function as a biocontrol agent. The classic example is Cryphonectria hypovirus 1 (CHV1), which infects *Cryphonectria parasitica* and reduces chestnut-blight canker development [7,8].

#### 2.2.2. Genetic Modification via Mycovirus-Derived VIGS System

Engineered virus-induced gene silencing (VIGS) extends natural mycovirus-mediated hypovirulence into a programmable system. A recombinant mycovirus can deliver sequence-specific RNA interference triggers that silence fungal virulence genes without modifying the fungal chromosome [19]. This approach directly addresses a central limitation of natural hypovirulence: the desired attenuation phenotype can be designed rather than merely discovered.

A key proof of concept used *Fusarium graminearum* gemytripvirus 1 (FgGMTV1), a tripartite circular ssDNA mycovirus, as a VIGS vector. Recombinant p26-D4 constructs expressing 75–150 bp target fragments persistently silenced fungal genes, including Tri101, which encodes a trichothecene 3-O-acetyltransferase that acetylates the C-3 hydroxyl of trichothecenes, thereby reducing their toxicity and contributing to fungal self-protection, and FgPP1, which is required for full development and pathogenicity [19].

Silencing Tri101 reduced deoxynivalenol (DON) production and virulence on wheat heads, whereas silencing FgPP1 impaired fungal colonization and symptom development [19].

Together, these constructs demonstrate that targeted gene silencing can generate attenuated strains with reduced virulence and reduced mycotoxin-associated risk.

The recombinant VIGS vectors remained stable during serial subculturing and were transmitted from hypovirulent donors to virulent recipients through hyphal contact under laboratory and field conditions. FgGMTV1-based VIGS therefore represents a programmable and transmissible form of engineered hypovirulence [19].

#### 2.2.3. Spontaneous or Induced Mutation and Phenotypic Attenuation

Mutation, gene deletion, phenotypic conversion, or prolonged subculturing can also reduce virulence while preserving traits useful for biological control. Such weakened strains may compete with virulent populations, occupy infection sites, induce host resistance, or produce antimicrobial metabolites [20,21]. *Ralstonia solanacearum*, for example, can undergo phenotypic conversion from fluidal to non-fluidal colonies with reduced virulence [22,23]. Takanashi et al. [24] similarly generated the hypovirulent *Calonectria ilicicola* strain UH2-1A by repeated subculturing of the virulent parental strain UH2-1; UH2-1A showed markedly reduced pathogenicity but retained normal mycelial growth and lacked obvious direct antagonism toward the parental strain.

#### 2.2.4. Phage-Associated Bacterial Attenuation

Bacteriophages can contribute to attenuation of bacterial plant pathogens, but direct phage-mediated killing must be distinguished from hypovirulence sensu stricto. Lytic phages that simply reduce bacterial density are biological control agents, whereas an attenuation framework applies when phage selection or persistent/lysogenic infection reduces pathogenicity while the bacterial host remains viable.

Selection for phage resistance can impose fitness costs on traits required for pathogenicity. In *R. solanacearum*, phage-selected resistant variants showed reduced in planta virulence associated with mutations affecting type IV pili, type II secretion, and quorum-sensing regulation [25]. A comparable phage-driven resistance–virulence trade-off has been demonstrated in *Burkholderia glumae*, where phage selection was associated with reduced virulence in rice seedlings [26]. These examples establish attenuation as an indirect evolutionary consequence of phage pressure rather than a consequence of pathogen killing alone.

Temperate or persistent phages may also modify bacterial phenotypes through lysogenic conversion, insertional effects, or phage-encoded regulators. However, direct agricultural evidence that such interactions can be deliberately and reproducibly exploited to generate stable attenuated biocontrol strains remains limited [27,28,29].

#### 2.2.5. Epigenetic Modification

Epigenetic regulation can modulate pathogen virulence without altering DNA sequence. In *C. parasitica*, CHV1 infection is associated with genome-wide changes in cytosine methylation, and comparative methylome analyses indicate broad hypovirus-associated perturbation of the fungal epigenome [30,31]. DNA methylation, histone modifications, and small RNAs can influence virulence-gene expression in phytopathogenic fungi and plant–fungus interactions [32,33]. These observations establish association between virus infection and epigenetic remodeling, but do not by themselves demonstrate that the epigenetic changes are the causal driver of hypovirulence.

Accordingly, direct evidence that epigenetic change alone generates a stable, field-useful hypovirulent phenotype remains limited. Epigenetic regulation is best viewed as a potential contributor to attenuation and phenotypic plasticity unless causation is demonstrated by targeted perturbation and phenotypic rescue or transfer experiments [34].

#### 2.2.6. Mild-Virus Attenuation for Cross-Protection

Mild plant–virus strains used for cross-protection arise naturally, through mutagenesis, or by targeted modification of viral virulence determinants. In Zucchini yellow mosaic virus (ZYMV), mutations in HC-Pro can attenuate symptoms while retaining infectivity sufficient to protect plants against severe strains [35,36]. This is not classical fungal hypovirulence: the protective agent is a mild virus persisting in the plant host. It is included here as a functionally related weakened-pathogen strategy because it uses controlled attenuation to prevent more damaging infection [37].

## 3. Key Case Studies and Comparative Lessons

Table 1 summarizes the case studies by disease, host, pathogen, attenuated agent, experimental setting, demonstrated benefit, retained protective competence, and principal limitation. The text below emphasizes comparative lessons rather than repeating every tabulated detail. We distinguish laboratory or controlled proof-of-concept studies, greenhouse evidence, field trials, and sustained operational deployment; a positive experimental result is not treated as evidence of reliable commercial performance. Four recurring archetypes are emphasized. First, CHV1 in chestnut blight illustrates self-propagating population-level attenuation, in which success depends on compatibility and transmission through the pathogen population. Second, SsHADV-1-infected DT-8 illustrates pathogen-to-endophyte conversion, showing that attenuation can transform a pathogenic interaction into a protective association. Third, subculture-derived UH2-1A of *C. ilicicola* illustrates non-viral attenuation, where loss of virulence is paired with retained growth competence and broad intraspecific protection. Fourth, avirulent bacteria and mild plant viruses illustrate functionally related systems in which attenuated agents protect mainly through niche occupation, immune activation, or cross-protection rather than classical mycovirus transmission. This organization addresses the central question of the review: which retained biological capacities, evidence levels, and ecological constraints determine whether attenuation becomes agriculturally useful?

### 3.1. Chestnut Blight: The Foundational Success Story

Chestnut blight, caused by the ascomycete *C. parasitica*, remains the foundational example of hypovirulence-based biocontrol. After its introduction into North America around 1904, the pathogen devastated American chestnut (*Castanea dentata*) populations, whereas European chestnut stands later showed spontaneous recovery in several regions [7,11].

Recovery in Europe was linked to transmissible hypovirulence in Italian chestnut stands [73] later associated with a dsRNA element [74] now known as Cryphonectria hypovirus 1 (CHV1) [75,76]. CHV1 spread through fungal populations and helped reduce disease impact in many European chestnut regions, establishing fungal virocontrol as a practical and conceptual model [10,75].

CHV1 is a positive-sense RNA mycovirus of approximately 12.7 kb that attenuates *C. parasitica* by altering pigmentation, asexual sporulation, female fertility, and, in some backgrounds, vegetative growth [8,77]. These effects reduce both local canker development and reproductive potential. In a defined field context, therapeutic application of CHV1-infected strains in Portuguese chestnut orchards achieved 90.3–93.8% canker healing after one year and 93.5–100% after four years in populations dominated by a single VC type [38]. These high values should therefore be interpreted as site- and population-specific field efficacy rather than as a universal performance estimate.

The key lesson from CHV1 is that attenuation alone is insufficient. In Europe, relatively low VC diversity facilitated hypovirus spread, whereas high VC diversity in eastern North America restricted transmission and limited population-level control [10,11,78,79]. Localized success in Michigan and Wisconsin shows that hypovirulence can work when virus–fungus compatibility, pathogen population structure, and ecological context align [80,81]. This case therefore provides the most established example of self-propagating population-level attenuation, but also demonstrates its central limitation: even a strongly attenuating virus can fail when transmission competence is constrained by local population structure.

### 3.2. Sclerotinia White Mold: From Pathogen to Endophytic Guardian

*Sclerotinia sclerotiorum* causes white mold or stem rot in more than 400 plant species. The hypovirulent strain DT-8 carries *S. sclerotiorum* hypovirulence-associated DNA virus 1 (SsHADV-1), a circular ssDNA mycovirus in the family Genomoviridae [39]. Unlike most mycoviruses, SsHADV-1 can infect healthy *S. sclerotiorum* hyphae extracellularly and can also be transmitted by the mycophagous fly *Lycoriella ingenua* [17,18].

DT-8 illustrates how attenuation can change a pathogen–plant interaction rather than merely reducing aggressiveness. In reported field trials, spraying DT-8 at early flowering reduced rapeseed stem-rot severity by 67.6% and increased yield by 14.9% [40]. Seed treatment established endophytic colonization in cereals; across multiple replicated field trials and locations, DT-8-treated wheat showed approximately 40–60% lower Fusarium head blight and 4–18% higher yield than untreated controls [43]. In dry bean, seed biopriming with SsHADV-1-infected local *S. sclerotiorum* isolates enhanced resistance to white mold, gray mold, and Rhizoctonia root rot under experimental conditions [41]. Together, these studies provide unusually strong field evidence for an attenuated fungal system, although they do not by themselves establish uniform commercial performance across crops, environments, and production systems.

### 3.3. Fusarium Species: Mycoviruses and Non-Pathogenic Strains

Fusarium species cause economically important diseases, including Fusarium head blight (FHB) in cereals and wilt diseases in many crops. Several mycoviruses have been associated with attenuation in Fusarium, but the most conceptually important recent advance is programmable hypovirulence.

Zhang, Wang, Ruan, Nzabanita, Wang and Guo [19] engineered an FgGMTV1-based VIGS vector to silence virulence-related genes in *F. graminearum*. In reported field proof-of-concept experiments, constructs targeting Tri101 and FgPP1 reduced FHB severity by approximately 72–76% and DON contamination by 21–40%, and recombinant viruses were transmitted horizontally through hyphal contact. This system demonstrates that attenuation can be rationally designed to suppress both disease and toxin-related food-safety risk, but it remains an experimental engineered platform rather than evidence of a commercially deployable product.

Other Fusarium examples remain earlier in the translational pipeline. CHV1 from *C. parasitica* can experimentally infect *F. oxysporum f. sp. lycopersici* and attenuate tomato wilt symptoms in controlled plant assays [44], demonstrating heterologous-host transfer but not field efficacy. HadV1-LA6 reduces growth and virulence of *F. oxysporum f. sp. cubense* in experimental assays [45], identifying a candidate for Panama-disease virocontrol whose transmission, stability, field efficacy, and non-target profile still require evaluation.

### 3.4. Verticillium Wilt: Hypovirulent Fungi as Immune Modulators

Verticillium wilt, caused mainly by the soil-borne fungus *V. dahliae*, severely affects cotton, olive, and other crops [82]. Because chemical control is limited and complete resistance is often unavailable, low-virulence Verticillium-related fungi provide useful models of host-mediated protection.

One *V. dahliae* strain co-infected with *V. dahliae* partitivirus 1 (VdPV1) and *V. dahliae* magoulivirus 1 (VdMoV1) showed inhibited mycelial growth, reduced conidial production, and attenuated virulence [47].

*Gibellulopsis nigrescens* CEF08111, isolated from healthy cotton, causes little disease but strongly protects cotton against *V. dahliae* [46]. Transcriptomic analysis indicates rapid activation of plant–pathogen interaction, MAPK, hormone-signaling, phenylpropanoid, and lignin-related defense pathways. Because these data show correlated host responses rather than direct causal dissection of each pathway, they are consistent with a substantial host-mediated component but do not establish that any single signaling pathway is necessary or sufficient for protection.

In olive, pre-inoculation with non-defoliating *V. dahliae* isolates protected cultivar ’Picual’ against highly virulent defoliating isolates, reproducing the natural recovery phenomenon observed in Mediterranean orchards [48].

Collectively, controlled Verticillium studies show that low-virulence fungi can provide protection through mycovirus-associated attenuation, pre-colonization, and host defense responses. However, the relative contribution of these mechanisms is system-specific, and broader field validation is still needed for stability, host range, application method, and consistency across environments.

### 3.5. Other Hypovirulent Fungal Pathogens

*C. ilicicola*, the causal agent of soybean red crown rot (RCR), Cylindrocladium black rot of peanut, and related root diseases, is difficult to manage because it persists in soil and crop residues [83]. Takanashi, Kobayashi and Jiang [24] generated UH2-1A by repeated subculturing of the virulent strain UH2-1. UH2-1A retained normal mycelial growth, showed markedly reduced pathogenicity, and lacked detectable direct antagonism toward UH2-1. In greenhouse pot experiments, mixtures containing UH2-1A at UH2-1A/UH2-1 ratios of ≥1 suppressed RCR, and protection extended to four additional virulent *C. ilicicola* isolates. This non-mycoviral system therefore demonstrates attenuation with retained growth competence and broad intraspecific protection. The protective mechanism, however, remains unresolved: the absence of obvious direct antagonism is consistent with a host-mediated component but does not establish it. Field efficacy, formulation, persistence, and phenotypic stability also remain to be tested.

*Rosellinia necatrix* causes white root rot in numerous woody and perennial crops [49]. Japanese surveys identified multiple mycoviruses with virocontrol potential. RnMBV1 causally reduced mycelial growth and virulence after introduction into virus-free strains [50], whereas RnPV1 effects appear context-dependent and may require interaction with other viruses [84,85,86]. Field studies in apple orchards showed that mycovirus introduction is feasible but dynamic, with additional viral elements acquired and disseminated in orchard populations [51]. RnHV2 also confers moderate hypovirulence in avocado isolates [52].

*Botrytis cinerea* causes gray mold on grapevine, strawberry, tomato, bean, and many other hosts. Several mycoviruses occur in Botrytis, and some virus-infected isolates show reduced virulence on grape berries or bean leaves, although attenuation varies among virus–fungus combinations [53,54].

In the rice blast fungus *M. oryzae*, MoCV1-A causes impaired growth, altered colony morphology, reduced pigmentation, suppressed conidial germination, and attenuated virulence in experimental systems [55,56,57]. Avirulent *M. oryzae* isolates can also induce blast resistance under controlled inoculation conditions [87,88,89]. The nonpathogenic field isolate AM16 carries mutations in Pmk1 and Mac1, genes required for conidiation, appressorium formation, and pathogenicity; complementation partially restores these traits [58,59,60]. This pathosystem therefore enables comparison of viral and mutation-derived routes to attenuation, but robust field performance of either approach remains to be established.

### 3.6. Bacterial Wilt: Avirulent Ralstonia as Plant Vaccines

The *R. solanacearum* species complex causes bacterial wilt in more than 250 plant species, including potato, tomato, banana, and peanut [23,90]. It can spontaneously convert during culture to non-fluidal variants with greatly reduced or absent virulence [22], making it a useful model for attenuated bacterial biocontrol.

Avirulent *R. solanacearum* strains can suppress disease through competition and host defense activation. In potato, avirulent cultures and even cell-free, heat-treated, or killed-cell preparations reduced wilt to varying degrees in controlled experiments [23]. In two reported tomato field trials, aRS combined with a microbial restoration substrate achieved control efficiencies of 80.79% and 85.79%, compared with lower control by aRS alone [61]. In tobacco, strain M4S reduced wilt and the effect was enhanced by a phage-containing filtrate [62]. FJAT1458 restricted virulent *R. solanacearum* colonization in tomato roots and induced PR-1a, GLUA, and CHI3 [63]. The evidence therefore supports both niche occupation and elicitor-mediated immunity, but performance remains dependent on strain, host, formulation, and environment.

### 3.7. Viral Cross-Protection: Mild Strains as Crop Vaccines

Mild-virus cross-protection is a functionally related form of attenuated-pathogen biocontrol. Since McKinney’s [91] early TMV observations, protective mild strains have been used against several major viral diseases. Mild CTV isolates have supported citrus production in regions affected by severe isolates [65,66]. PRSV mild strains such as HA 5-1 were deployed in field programs in Taiwan, although protection declines against genetically divergent isolates [67,68,69]. PepMV mixtures have been evaluated in greenhouse tomato [70,71], whereas ZYMV-WK has field evidence and selected HC-Pro mutants have shown high protection in controlled challenge experiments [35,36,72]. Mechanistically, cross-protection involves superinfection exclusion, viral protein-mediated interference, replication-level competition, and homology-dependent RNA silencing [37,92,93]. Unlike fungal mycovirus systems, the attenuated agent resides in plant tissues rather than in a pathogen population. Its usefulness therefore depends on controlled symptom expression, persistence, genetic stability, and sufficient relatedness to the challenge virus.

## 4. Mechanisms of Hypovirulence-Based Protection

Across fungal, bacterial, and viral systems, attenuation arises through different molecular mechanisms. The commonality is therefore not a shared cause of weakened virulence, but a shared functional architecture: reduced disease-causing capacity coupled with retention of at least one protective competence. We refer to this as the attenuation–competence balance. Depending on the pathosystem, the retained competence may be transmission, host colonization, ecological competition, host immune stimulation, or superinfection exclusion. The four categories below are functional comparison levels, not claims that fungal hypovirulence, bacterial attenuation, and viral cross-protection are mechanistically equivalent. Figure 1 summarizes this framework.

### 4.1. Population-Level Attenuation

Population-level attenuation occurs when transmissible hypovirulence determinants spread through a pathogen population and convert virulent individuals into less virulent ones. In filamentous fungi, this typically requires hyphal anastomosis between compatible individuals, enabling cytoplasmic exchange of mycoviruses or other elements [78]. Its success depends on both attenuation strength and transmission competence within the local pathogen population, making pathogen population structure a central determinant of field efficacy.

### 4.2. Direct Antagonism and Altered Pathogen Physiology

At the pathogen-physiology level, attenuating elements can disrupt signaling, development, reproduction, toxin production, or metabolism. CHV1 in *C. parasitica* reprograms fungal gene expression, perturbs G-protein-regulated cAMP signaling, alters pigmentation and reproductive development, and down-regulates genes such as cryparin, contributing to reduced canker development [8,94].

Toxin suppression is especially important for food-safety pathogens. In *F. graminearum*, FgV1-SD4 infection reduced virulence and eliminated detectable DON in harvested wheat seeds in the reported experiment [95]. In *S. sclerotiorum*, the SsHADV-1 replication-associated protein reduces oxalic acid production and oah1 expression, linking a viral factor to attenuation of a major pathogenicity determinant [96,97].

Attenuation can also reprogram metabolism in ways that affect microbial competition. In *S. sclerotiorum* strain SCH767, which harbors six mycoviruses, hypovirulence was associated with a more than 10-fold increase in 2-ethyl-1-hexanol, a volatile compound with antifungal, antibacterial, and nematicidal activity [98]. Thus, weakened pathogens may sometimes gain traits that suppress neighboring microorganisms while losing aggressiveness.

These examples show that direct physiological attenuation is not simply “pathogen weakening.” It can also reshape metabolism, competition, and toxin-related risk, thereby linking individual pathogen physiology to population-level disease suppression.

### 4.3. Plant Defense Activation, Priming, and Endophytism

Plant defense priming is a broad physiological state in which prior stimulation enables a faster or stronger response to subsequent challenge; it should not be used interchangeably with systemic acquired resistance (SAR) or induced systemic resistance (ISR). SAR classically follows local infection and is strongly associated with salicylic acid (SA), NPR1, and systemic expression of pathogenesis-related genes, whereas classical ISR is typically elicited by beneficial microbes and often depends on jasmonic acid (JA)- and ethylene (ET)-regulated signaling without constitutive activation of the same SAR markers [99,100,101,102,103,104,105]. Historical reports that weakened pathogens could ‘immunize’ plants [5,6,99] are therefore best interpreted as early observations of induced protection rather than evidence for a specific modern signaling category.

Hypovirulent or attenuated endophytes may activate basal defense, generate a primed state, or engage signaling that overlaps only partly with canonical SAR or ISR. In dry bean, SsHADV-1-infected *S. sclerotiorum* strains induced constitutive expression of PR1 and PR2 and enhanced defense-gene responsiveness after pathogen challenge [41]. In rapeseed, DT-8 reprogrammed ROS-, phenylpropanoid-, brassinin-, and hormone-associated pathways while maintaining endophytic persistence [40,106].

SsHADV-1-infected DT-8 therefore demonstrates a pathogen-to-endophyte transition: attenuation permits persistence with limited disease, while the colonized plant gains enhanced resistance to virulent *S. sclerotiorum* [40]. This outcome is consistent with defense activation and priming, but should not be labeled as canonical SAR or ISR unless the defining systemic and genetic requirements are experimentally established.

Dual RNA sequencing indicates that DT-8 colonization is an active, balanced interaction. Host defense pathways involving ROS, phenylpropanoid metabolism, and brassinin biosynthesis are activated, while DT-8 expresses effector-like proteins, detoxification enzymes, and antioxidant components that may moderate host defenses sufficiently for endophytic accommodation [106]. Similar host-mediated protection occurs in dry bean, where SsHADV-1-infected *S. sclerotiorum* strains colonize endophytically and enhance resistance to white mold, gray mold, and Rhizoctonia root rot [41].

A related cotton system involves the hypovirulent endophyte *G. nigrescens* CEF08111, which protects against *V. dahliae* without obvious direct antagonism [46]. Associated responses include MAPK and hormone signaling, SA/JA accumulation, calcium signaling, cell-wall metabolism, and phenylpropanoid/lignin biosynthesis. These associations support a model of host-mediated defense activation and/or priming, but causal pathway requirements remain to be tested.

### 4.4. Competitive Exclusion and Cross-Protection

Bacterial and plant–virus systems usually lack mycovirus-like population conversion. Instead, they rely more strongly on pre-emptive colonization, niche occupation, elicitor-mediated defense activation, molecular interference, or superinfection exclusion. They are included here because they test the same functional principle: a mild or attenuated agent prevents subsequent damage by a more virulent counterpart.

#### 4.4.1. Bacterial Hypovirulence: Niche Exclusion and Immune Priming

In bacterial wilt, avirulent or genetically attenuated *R. solanacearum* strains can protect through vascular niche competition and immune stimulation. T3SS-defective mutants, for example, can still colonize roots and xylem but lose pathogenicity; in tomato, an HrcV mutant restricted subsequent colonization by a pathogenic strain, with individual xylem vessels often occupied by either the mutant or the virulent strain [64].

Defense activation also contributes. *Arabidopsis* pre-inoculated with a ΔhrpB mutant showed reduced multiplication of virulent *R. solanacearum* and extensive host transcriptional reprogramming [107]. In potato, avirulent cultures, supernatants, boiled preparations, and killed cells all reduced wilt to varying degrees and altered phenolic, PPO, and POD responses [23]. These findings indicate that attenuated Ralstonia protection combines niche occupation with MAMP- or elicitor-mediated defense priming, rather than reflecting simple physical exclusion [90,108].

#### 4.4.2. Viral Cross-Protection: Three Overlapping Mechanisms

Mild-virus cross-protection occurs when prior infection by a mild strain restricts a later, closely related severe strain. Superinfection exclusion is important, but no single mechanism explains all cases; current evidence supports overlapping viral protein-mediated interference, homology-dependent RNA silencing, and replication-level competition [37,93,109,110].

Viral proteins can mediate interference. TMV coat-protein studies showed that viral protein expression can delay subsequent infection [111,112,113], and in CTV, p33 is required for whole-plant exclusion of closely related challenge viruses, although cellular exclusion can occur independently [114,115].

RNA silencing provides a sequence-specific layer of protection. Virus-derived siRNAs produced during mild-strain infection can guide ARGONAUTE-containing complexes against homologous viral RNAs, explaining why protection is often strongest between closely related strains. Ratcliff et al. [116] showed that RNA-mediated cross-protection is functionally equivalent to post-transcriptional gene silencing, although sequence identity alone cannot reliably predict protection across systems [37,93,116].

A third layer is replication-level competition or exclusion. Once a primary virus establishes replication complexes and occupies host resources, a related challenge virus may be unable to establish efficiently [109,110]. The required interval between protective and challenge inoculation therefore varies among virus–host combinations.

Together, viral cross-protection illustrates the same attenuation–competence balance in a viral context: the mild strain must cause limited damage yet persist long enough, and be similar enough to the challenge strain, to establish a protective antiviral state.

## 5. Challenges and Limitations

Despite substantial progress, field deployment of attenuated-pathogen biocontrol remains limited by biological, ecological, regulatory, and technical constraints. For clarity, the following sections distinguish constraints demonstrated experimentally (for example, vegetative-incompatibility barriers and temperature-dependent phenotypes) from plausible or theoretical risks that require prospective assessment (for example, non-target spread, recombination, or genetic reversion).

### 5.1. Vegetative Incompatibility: A Major Bottleneck to Mycovirus Transmission

Most mycoviruses lack efficient extracellular transmission and spread horizontally mainly through hyphal anastomosis or vertically through spores [14,117]. Incompatibility between donor and recipient strains can trigger localized cell death and restrict cytoplasmic exchange, reducing virus transmission [79] In *C. parasitica*, six vic loci can generate 64 VC genotypes, and virus transmission is highest between identical genotypes but often declines when one or more vic loci differ [78,79,118]. High VC diversity can therefore prevent a strongly hypovirulent virus from becoming self-sustaining in the field.

SsHADV-1 is a notable exception because purified particles can infect *S. sclerotiorum* extracellularly across vegetative compatibility groups and can also use the mycophagous insect *L. ingenua* as a vector [17,18]. Such alternative transmission routes greatly improve biocontrol potential but remain unusual among characterized mycoviruses.

### 5.2. Environmental Variability and Consistency of Efficacy

Mycovirus-associated phenotypes emerge from interactions among viral genotype, fungal background, plant host, microbiota, and environment [119,120,121]. Different CHV1 strains introduced into distinct *C. parasitica* backgrounds produce different hypovirulence phenotypes [121], and CHV1–*C. parasitica* interactions show significant virus genotype × fungal genotype × temperature effects [119].

Temperature affects fungal growth, viral replication and accumulation, transmission, and phenotype expression. CHV1 accumulation in *C. parasitica* varies from 5 to 30 °C, with generally higher levels at 15–25 °C and stronger growth inhibition at 20–25 °C [122]. Thermotherapy can eliminate some mycoviruses but not others, indicating that thermal stability is system-specific [123,124].

These genotype × environment interactions create a gap between laboratory efficacy and field reliability [14]. Early establishment may help: DT-8 seed biopriming colonized rapeseed systemically, reduced stem rot, increased yield, and altered microbial networks in field experiments conducted across two locations and three years [42], while DT-8-treated wheat reduced FHB by approximately 40–60% and increased yield by 4–18% across replicated trials at multiple locations and seasons [43]. These are encouraging examples of field robustness, but they do not establish universal performance; broad multi-location, multi-season, genotype-diverse, and formulation-specific trials remain essential.

### 5.3. Genetic and Phenotypic Stability

Long-term stability is critical. Demonstrated sources of variation include mycovirus titer and transmission differences among fungal genotypes, developmental stages, and environments [13,14], and loss or marked decline of a hypovirulence-associated virus can weaken the protective phenotype. Non-viral attenuated strains and mild plant viruses are also subject to evolutionary change during propagation and field persistence, although the probability and consequences are system-specific.

For cross-protective plant viruses, attenuation based on a small number of mutations creates a plausible risk of reversion or compensatory evolution. ZYMV HC-Pro mutants can provide strong protection [35,36], but construct design alone cannot establish long-term stability. Serial passage, whole-genome monitoring, host-range testing, phenotypic re-evaluation, and long-term field surveillance are therefore appropriate tools for distinguishing demonstrated stability from theoretical reversion risk before deliberate deployment.

### 5.4. Biosafety and Ecological Considerations

Intentional release of self-replicating viruses or hypovirulent microorganisms requires a tiered biosafety assessment. A demonstrated constraint is that natural mycovirus transmission often depends on fungal compatibility; experimental transfer can nevertheless reveal a broader potential host range than is evident from natural occurrence [14,15]. SsHADV-1, for example, infects several Sclerotinia species and the insect *L. ingenua* but did not infect more distantly related fungi tested in the original study [17,18]. Non-target risk should therefore be measured rather than inferred, using defined host-range panels, recovery or qPCR-based persistence assays, and where appropriate contained microcosm studies before field release.

Other concerns—including dispersal beyond treated sites, recombination, evolutionary compensation, effects on microbial community structure, and selection for pathogen genotypes that block transmission—are biologically plausible but are not equally demonstrated across systems. They should be evaluated with system-specific monitoring: spatial sampling of the agent and attenuating element, serial genomic analysis, comparison of treated and untreated microbiomes, and multi-season assessment of persistence and phenotype. For toxin-producing pathogens such as *F. graminearum*, attenuation must also be accompanied by direct mycotoxin measurement; the engineered FgGMTV1 system is encouraging because both FHB severity and DON contamination were reduced in reported field proof-of-concept experiments [19].

### 5.5. Technical, Regulatory, and Economic Barriers

Practical deployment requires scalable production, formulation, storage, delivery, and quality control. Many mycoviruses cannot be propagated independently of their fungal hosts, and the lack of extracellular transmission in most systems complicates formulation. Shelf life, fungicide compatibility, inoculum stability, application method, dose, timing, recovery of the agent from the target niche, and performance across locations and seasons should be established before commercialization [14].

Regulation is also challenging because host specificity, non-target effects, persistence, genetic stability, and dispersal remain incompletely understood for many candidates. Recent reviews identify transmission, formulation, environmental stability, biosafety, and regulatory data requirements as major barriers between discovery and commercialization [15]. Hypovirulence-based control should therefore be positioned as one component of IPM rather than as a universal replacement for host resistance, biological amendments, or conventional disease management. Non-mycoviral attenuation may offer complementary routes: UH2-1A suppressed soybean red crown rot caused by multiple virulent isolates while retaining normal mycelial growth [24], but its practical value will depend on scalable production, shelf stability, field efficacy, persistence, and compatibility with existing IPM practices.

## 6. Future Directions and Emerging Technologies

Emerging technologies may strengthen attenuated-pathogen biocontrol by improving design, discovery, delivery, and ecological performance, but their evidence status differs substantially. Experimentally demonstrated examples include FgGMTV1-based VIGS in *F. graminearum* [19] and extracellular infection of *S. sclerotiorum* by SsHADV-1 [17]. By contrast, combining hypovirulent agents with synthetic communities, advanced biomaterials, or other delivery platforms remains prospective unless specifically validated. The goal is not simply to find weakened pathogens, but to rationally optimize the attenuation–competence balance while clearly separating demonstrated applications from proposed research directions.

### 6.1. Synthetic Biology and Engineered Hypovirulence

Synthetic biology can make hypovirulence more programmable. Extracellularly infectious SsHADV-1 shows that transmission barriers are not universal [15,17], while FgGMTV1-based VIGS demonstrates that mycoviruses can be engineered to silence fungal virulence genes and generate transmissible hypovirulent strains [19].

In that system, targeting Tri101 or FgPP1 converted virulent *F. graminearum* into attenuated strains that suppressed FHB and mycotoxin contamination in field proof-of-concept experiments [19]. This establishes the feasibility of engineered mycovirus-mediated attenuation, but not the commercial feasibility, long-term environmental stability, or regulatory acceptability of such a product.

Related opportunities exist in bacterial and viral systems. Avirulent *R. solanacearum* can combine competition with defense induction [63], and ZYMV HC-Pro mutants illustrate rational attenuation of plant viruses for cross-protection [36]. These examples support design strategies that consider virulence, ecological competence, stability, and immune-stimulating capacity together.

### 6.2. Cross-Kingdom RNA Interference and Programmable Gene Silencing

RNA interference provides experimentally established tools for selective gene suppression, but not every RNA-based crop-protection technology constitutes hypovirulence. Cross-kingdom small-RNA trafficking is important in plant–fungus interactions: *B. cinerea* can deliver small RNAs that suppress host immunity [33], whereas plant-expressed or topically applied dsRNAs can silence fungal genes through HIGS or SIGS [125]. Mycovirus-mediated VIGS differs in that the silencing vector replicates within the fungal pathogen [19].

Natural mycovirus-mediated hypovirulence and engineered mycovirus VIGS are direct attenuation strategies, whereas HIGS and SIGS are conceptually related RNA-based control technologies. Combining these approaches to reinforce attenuation, improve transmission, or overcome compatibility barriers remains a research direction that requires direct experimental validation.

### 6.3. Microbiome Engineering and Synthetic Consortia

Hypovirulent agents act within plant-associated microbial communities. Rhizosphere, phyllosphere, and endosphere microbes can influence pathogen establishment, host immunity, resource competition, and persistence of introduced agents. Microbiome engineering may therefore improve consistency, but direct demonstrations of designed synthetic communities containing hypovirulent pathogens remain very limited; this subsection should be read as a prospective research direction rather than an established application.

Synthetic microbial communities can combine taxa with complementary functions such as antibiosis, niche exclusion, resource competition, growth promotion, and induced resistance [126]. For hypovirulence-based systems, rational partners could suppress residual virulent populations or reinforce host defense while the attenuated pathogen occupies its ecological niche.

This remains a promising but largely future-oriented direction. Network analysis, community profiling, and controlled SynCom experiments will be needed to identify compatible partners and test whether consortia improve persistence and reliability under field conditions.

### 6.4. Next-Generation Mycovirus Discovery and Functional Screening

High-throughput sequencing has rapidly expanded known mycovirus diversity. For example, analysis of 248 *B. cinerea* field isolates identified 92 mycoviruses, including 62 putative new species [127], and broader metatranscriptomic resources continue to reveal new fungal viruses [15].

The bottleneck is now functional validation rather than discovery. Because most mycoviruses do not cause obvious hypovirulence, candidates must be introduced into virus-free fungal backgrounds and evaluated for effects on growth, sporulation, stress tolerance, transmission, toxin production, and pathogenicity.

Future pipelines should integrate metatranscriptomics, comparative genomics, infectious clones or transfection systems, and high-throughput phenotyping. Once sufficiently large validated datasets exist, computational models may help prioritize viruses with desirable attenuation and transmission traits.

### 6.5. Precision Delivery and Formulation Science

Field application depends on stable and practical delivery. SsHADV-1 provides direct proof that purified viral particles can infect *S. sclerotiorum* extracellularly on fungal hyphae or plant surfaces and reduce disease [17]. This unusual property makes SsHADV-1 a useful proof of principle for formulation, but it should not be generalized to the many mycoviruses that lack an extracellular infectious phase.

Biomaterials and RNA-delivery technologies provide transferable concepts rather than direct evidence for hypovirulence products. Layered double-hydroxide clay nanosheets can protect topically applied dsRNA, provide sustained release, and prolong RNAi-mediated antiviral protection in plants [128]. Similar formulation principles might eventually improve delivery of extracellularly infectious mycoviruses, fungal propagules, or hypovirulence-associated materials, but such applications have not yet been directly demonstrated.

Seed coating may be useful for attenuated strains capable of endophytic establishment, whereas localized lesion treatment remains relevant for perennial crops such as chestnut. Technologies such as long-term encapsulated mycovirus storage or drone-mediated canker inoculation should be treated as conceptual until experimentally validated.

Overall, the field is moving from opportunistic discovery toward rational design of attenuated-pathogen systems. The most promising approaches will optimize virulence attenuation, transmission or colonization competence, ecological persistence, host immune priming, safety, and deliverability as integrated traits.

## 7. Conclusions

Hypovirulence-based biocontrol has developed from a largely serendipitous phenomenon into a collection of experimentally tractable crop-protection strategies. Evidence ranges from long-standing field use and population-level suppression in the CHV1–chestnut system, through multi-location field trials of SsHADV-1-associated DT-8, to greenhouse or laboratory proof-of-concept studies for heterologous mycovirus transfer, non-viral attenuation, and engineered systems. These evidence levels should not be conflated when judging practical readiness.

The comparative contribution of this review is the attenuation–competence framework: mechanistically distinct fungal, bacterial, and viral systems are evaluated according to what protective capacity is retained after attenuation, how strong the supporting evidence is, and which limitation is most likely to block translation. The framework therefore does not redefine all weakened pathogens as mechanistically equivalent forms of hypovirulence; rather, it provides a common functional basis for comparing transmission, colonization, competition, immune stimulation, and cross-protection across pathosystems.

Future progress will depend on integrating natural hypovirulence with programmable gene silencing, mycovirus discovery, formulation science, and rigorous biosafety evaluation while testing prospective approaches such as microbiome engineering before claiming practical feasibility. Rather than replacing existing control measures, attenuated-pathogen biocontrol is most likely to become a specialized component of IPM where attenuation is stable, protective competence is retained, and field delivery is reliable.

## Figures and Tables

**Table 1:** Representative hypovirulence-based disease-control systems in agricultural pathosystems. Experimental setting, demonstrated benefit, retained protective competence, and principal translational limitation are indicated to distinguish evidence level from practical readiness.

Disease Name	Host Plant	Pathogen Name	Hypovirulent Strain and Origin	Experimental Setting/Demonstrated Benefit	Mechanism/Retained Competence/Principal Limitation	References
Chestnut blight	European and American chestnut	*Cryphonectria parasitica*	CHV1-infected hypovirulent strains; naturally disseminated or therapeutically introduced	Field/orchard evidence: canker healing exceeded 90% at specific Portuguese sites under favorable VC conditions; natural spread contributed to European recovery	Population-level attenuation via hyphal transmission. Main limitation: vegetative incompatibility and local VC diversity can prevent virus spread; efficacy is strongly context-dependent	[7,8,10,11,38]
Sclerotinia white mold/stem rot	Rapeseed, dry bean, and other dicot crops	*Sclerotinia sclerotiorum*	SsHADV-1-infected strain DT-8 and related local virus-infected isolates	Field evidence in rapeseed; experimental/greenhouse evidence in dry bean. Reported benefits include reduced Sclerotinia stem rot, increased rapeseed yield, and enhanced resistance to white mold, gray mold, and Rhizoctonia root rot after biopriming.	Extracellular virus transmission, endophytic persistence, and host defense activation. Main limitation: crop/strain/environment dependence; formulation and broad field reproducibility require further validation.	[17,18,39,40,41,42]
Fusarium head blight	Wheat	*Fusarium graminearum*	SsHADV-1-infected *Sclerotinia sclerotiorum* strain DT-8 used as an endophytic biocontrol agent	Replicated field evidence: seed treatment established endophytic colonization and reduced FHB by ~40–60% while increasing yield by 4–18% across multiple trials.	Protection is associated with endophytic establishment and host-mediated disease suppression rather than attenuation of *Fusarium graminearum*. Main limitation: performance depends on crop, environment, persistence, and application method.	[43]
Fusarium head blight	Wheat	*Fusarium graminearum*	Engineered FgGMTV1-based VIGS constructs p26-D4-Tri101 and p26-D4-FgPP1	Field proof-of-concept: FHB severity reduced by ~72–76% and DON contamination by 21–40% in the reported experiments	Programmable, transmissible VIGS targeting virulence/mycotoxin genes. Main limitation: engineered-system stability, transmission efficiency, biosafety and regulatory requirements; no commercial validation	[19]
Fusarium wilt	Tomato	*F. oxysporum f. sp. lycopersici*	CHV1 experimentally transferred from *C. parasitica* to *F. oxysporum*	Controlled plant assay: CHV1-infected strains caused only mild symptoms and showed reduced vascular colonization	Heterologous mycovirus infection demonstrates cross-host attenuation potential. Main limitation: experimental transfer only; field transmission, stability and efficacy are untested	[44]
Fusarium wilt/Panama disease	Banana	*F. oxysporum f. sp. cubense*	Hadaka virus 1 isolate HadV1-LA6	Laboratory/controlled pathogenicity assays: reduced fungal growth and virulence	Mycovirus-associated attenuation. Main limitation: transmission, genetic stability, non-target effects and field efficacy remain to be established	[45]
Verticillium wilt	Cotton	*Verticillium dahliae*	Protective endophytic fungus *Gibellulopsis nigrescens* CEF08111, isolated from healthy cotton	Controlled plant/transcriptome study: strong disease protection with minimal symptoms and broad activation of defense-associated pathways	Protection is consistent with host defense activation and structural reinforcement. Main limitation: pathway causality and field consistency have not been established	[46]
Verticillium wilt	Cotton/olive and related hosts	*Verticillium dahliae*	Co-infection with VdPV1 and VdMoV1; non-defoliating low-virulence *V. dahliae* isolates	Controlled studies: mycovirus co-infection reduced growth, conidiation and virulence; pre-inoculation with low-virulence isolates protected olive against defoliating isolates	Mycovirus-mediated attenuation or protective pre-colonization. Main limitation: system-specific effects and limited evidence for consistent field deployment	[47,48]
Red crown rot	Soybean	*Calonectria ilicicola*	UH2-1A generated by repeated subculturing of virulent strain UH2-1	Greenhouse pot assays: UH2-1A suppressed RCR caused by UH2-1 and four additional virulent *C. ilicicola* isolates	Non-mycoviral attenuation with retained growth competence. Mechanism remains unresolved; absence of direct antagonism is consistent with, but does not establish, host-mediated protection. Main limitation: no field validation	[24]
White root rot	Apple, avocado, grapevine and other woody crops	*Rosellinia necatrix*	RnMBV1, RnHV2 and other mycovirus-infected strains	Laboratory pathogenicity evidence plus orchard studies demonstrating feasibility and complexity of virus introduction	Mycovirus-mediated attenuation. Main limitation: fungal compatibility, transmission efficiency and dynamic coinfection with multiple viruses can affect outcome	[49,50,51,52]
Gray mold	Grapevine, strawberry, tomato, bean and other hosts	*Botrytis cinerea*	Botybirnavirus-, mitovirus- and other RNA-virus-infected isolates	Laboratory/detached-tissue studies: some virus-infected isolates show reduced virulence on grape berries or bean leaves	Virus–fungus-specific attenuation; altered infection-cushion formation in some systems. Main limitation: variable phenotype and very limited field evidence	[53,54]
Rice blast	Rice	*Magnaporthe oryzae*	MoCV1-A-infected isolates; avirulent field isolate AM16	Laboratory/controlled plant evidence: MoCV1-A reduces fungal growth/conidial germination and virulence; AM16 induces blast resistance	Mycovirus-associated attenuation or mutation-derived avirulence affecting development/pathogenicity. Main limitation: field efficacy, stability and delivery remain unestablished	[55,56,57,58,59,60]
Bacterial wilt	Tomato, potato, tobacco and other solanaceous crops	*Ralstonia solanacearum species complex*	Avirulent or phenotypically converted *R. solanacearum* strains, including FJAT1458 and M4S	Controlled studies plus reported tomato field trials: reduced wilt; some treatments improved yield or achieved high trial-specific control	Retained niche competence plus elicitor-mediated defense activation. Main limitation: strain/host/environment dependence and biosafety/persistence of live attenuated bacteria	[22,23,61,62,63,64]
Citrus tristeza	Citrus	Citrus tristeza virus	Mild/protective CTV isolates	Long-term orchard deployment in several citrus-growing regions has supported production under pressure from severe isolates	Persistent mild infection and cross-protection/superinfection exclusion. Main limitation: protection is strongly strain/genotype-dependent and may not block divergent challenge isolates	[65,66]
Papaya ringspot	Papaya	Papaya ringspot virus	Nitrous-acid-induced mild strains HA 5-1, HA 6-1 and recombinants	Large-scale field deployment in Taiwan produced substantial protection and yield benefits in susceptible papaya	Mild-virus persistence; sequence-related cross-protection and RNA silencing contribute. Main limitation: protection can fail against genetically divergent isolates	[67,68,69]
Pepino mosaic	Tomato	Pepino mosaic virus	Attenuated VX1 and VC1 isolates; mixed-genotype products	Greenhouse studies/deployment: mild isolates protected tomato; mixtures broadened protection against divergent PepMV genotypes	Multi-genotype exclusion/interference. Main limitation: genotype matching, timing and stability of the protective infection determine efficacy	[70,71]
Zucchini yellow mosaic	Cucumber, melon, squash and other cucurbits	Zucchini yellow mosaic virus	ZYMV-WK and engineered/selected HC-Pro mutants	ZYMV-WK has field evidence for improved marketable yield; selected HC-Pro mutants provided ~84–100% protection in controlled challenge experiments	Attenuated mild-virus infection; RNA-silencing and exclusion mechanisms contribute. Main limitation: strain matching and genetic/phenotypic stability, including reversion risk	[35,36,72]

## Data Availability

No new data were created or analyzed in this study. Data sharing is not applicable to this article.
